# Supplementary material for: Are carbon emissions trading and green financial instruments synergistic? -Comprehensive quantitative research based on content analysis
Source: PLoS One. 2024 Mar 7;19(3):e0298601. doi: 10.1371/journal.pone.0298601 (PMC10919601; doi:10.1371/journal.pone.0298601)
Supplement: S1 Data — (ZIP) [file pone.0298601.s001.zip › Data/地级市Z_F工作报告.zip!/╕╩╦α/░╫╥°╩╨/└┤╫╘╣1⁄2╓┌║┼í╛┬φ┐╦╩2╛▌═°í┐_2014.docx]

各位代表：

现在，我代表市人民政府，向大会作工作报告，请予审议，并请政协委员及列席人员提出意见。

2013年工作回顾

2013年，在省委、省政府和市委的正确领导下，全市上下认真贯彻党的十八大及十八届二中、三中全会精神，积极应对经济下行压力加大的严峻形势，牢牢把握 稳中求进、好中求快的总基调，一手抓转型发展，一手抓民生保障，扎实推进稳增长、调结构、促改革、惠民生各项工作，较好完成了市八届人大二次会议确定的主 要预期目标。全市完成生产总值464.49亿元，同比增长12.8%；工业增加值212.86亿元，增长15.7%；固定资产投资421.58亿元，增长 31.38%；社会消费品零售总额135.75亿元，增长14.8%；公共财政预算收入24.11亿元，增长20.63%；居民消费价格总水平涨幅 2.5%；城镇居民人均可支配收入18279.7元，增长11.5%；农民人均纯收入5140.1元，增长14.3%；城镇登记失业率2.46%；人口自 然增长率5.89‰。

1.农业经济活力增强。实现农业增加值55.01亿元，增长6.5%。粮食生产实现“十连增”，总产量达到77.76万吨，同比增长7.85%。特色优势 产业快速发展，新改扩建日光温室和塑料大棚1.65万亩，瓜菜种植面积达到97万亩；新建标准化养殖小区75家，牛、羊饲养量分别达到17.23万头、 430.15万只；新增特色经济林15万亩。农业科技推广力度不断加大，推广全膜双垄沟播131.13万亩，高效节水示范灌溉91.58万亩，农作物良种 覆盖率、畜禽良种普及率分别达到95%、90%以上。农村基础设施建设扎实推进，引洮一期会宁北部供水、靖会工程甘沟干渠改扩建、兴电灌区齐家大岘除险加 固、双永供水等重点工程加紧建设，双永灌区等土地整理开发项目全面完成，新增有效灌溉面积2.5万亩，兴修梯田15.55万亩，完成水土保持综合治理 174.37平方公里。农业组织化程度进一步提升，新增龙头企业14家，总数达到331家，农产品加工转化率达到41%；新增农民专业合作社450家，总 数达到2508家，居全省首位；扶持土地流转经营大户55户，土地流转面积40.63万亩。会宁县被评为“中国亚麻籽之乡”，银特番茄酱等4个产品获十一 届中国国际农产品交易会金奖。

2.工业经济稳中有升。实现规模以上工业增加值195.13亿元，增长16%。主导产业集聚效应逐渐显现，3个千亿元产业链增加值占GDP比重 35.5%。白银公司铜冶炼渣综合利用、寿鹿山新型干法水泥、刘化一期15万吨硝酸、长通铜铝导体新型材料、中天化工无水氟化铝、中电投红山风电场一期、 中电国际上沙沃光伏发电等重大项目建成投产；锌冶炼资源综合利用、热电联产、鸿泰铝合金产业链、北方三泰氯碱、东方钛业钛白粉、凯斯瓷业等项目进展顺利。 靖煤集团整体重组刘化集团，成为国有资产结构调整、优化组合的成功典范。白银矿田深部及外围找矿取得重大突破，新增铜及多金属资源量约50万吨,被列为国 家级地质找矿整装勘查区。企业自主创新能力不断增强，昌源化工重铬酸钠制取技术达到国际领先水平，长通超导电缆导体用于法国国际热核聚变试验堆和神舟系列 飞船；郝氏炭纤维公司成为行业唯一全产业链企业，被列为省级工程技术研究中心；“容和”、“条山”两个商标被认定为“全国驰名商标”；中集华骏、甘肃稀土 等4家企业被认定为国家知识产权优势企业；引进高层次急需紧缺人才12名，新增高新技术企业3家、省级重点实验室1家、省级工程技术研究中心1家，全市专 利申请连续5年增幅30%以上，万人发明专利拥有量达到0.64件，我市被列为国家知识产权试点城市和省级创新型试点城市，继2003年后再次荣获全国科 技进步先进市。

3.第三产业持续发展。完成增加值155.92亿元，增长10.8%。文化旅游业不断融合。全市文化企业达到666个，完成文化产业增加值5.1亿元，同 比增长47.83%；建成基层文化大院61个，成功举办第十三届PHE国际青少年书画大赛，市第三届凤凰文艺奖揭晓，爱乐合唱团在维也纳世界合唱比赛中获 表演水平A级，舞蹈《背鼓子》代表甘肃亮相央视“直通春晚”节目，秦腔剧《靖远起义》荣获省第七届敦煌文艺奖，纪录片《大会师》、电影《爱在心里》在我市 拍摄完成，忠恒文化、陇烨陶瓷公司入选全省首批百家骨干文化企业。全市旅游规划和黄河风情线旅游规划编制完成，黄河假日城、龙湾古镇改造、法泉寺、雪山 寺、寿鹿山等景区建设加快推进，第三届乡村旅游节、第五届红色旅游节成功举办，会宁被评为“中国最佳红色旅游示范城市”；新增“农家乐”经营户140户， 市旅游服务中心和旅游商品销售中心启动运营；全年旅游接待人数501.7万人次，同比增长35.6%，旅游收入27.32亿元，同比增长37.4%。商贸 服务业加快发展。编制完成市场体系建设、现代物流和再生资源产业发展规划；白银公司现代工业物流园、中进大西北仓储物流园等核心园区建设启动，义乌商贸 城、靖远浙江商贸城、银西汽车商贸服务城、忠恒金地年鲜综合农贸市场等项目加紧建设，金利苑农贸市场、甘肃品高万吨冷链项目建成投运，国芳国际、星光银座 等商贸综合体项目基本建成；改造农贸市场12处，改扩建乡镇商贸配送中心7家、农家店260个。金融服务业全面提升。白银城投债成功发行，直接融资13亿 元；全市小贷公司发展到31家，累计发放贷款13.66亿元，15户担保机构发生担保额21.5亿元；创建“信用村”14个；发放“三权”抵押贷款 3162万元，发放妇女小额、双联惠农、牛羊蔬菜贷款21.36亿元；全市银行存贷比增加2.85个百分点。甘肃欣庆环保科技等6家公司在上海、深圳前海 股权交易中心挂牌交易。非公经济实力增强。全市非公经济市场主体达到59370户，比上年增加13933户，注册资金首次超过公有经济，预计增加值增长 17.2%，总量占全市GDP比重达到44%。

4.重点项目强力推进。全市在建项目1012个，同比增加241个。全年实施招商引资项目362个，到位资金293.8亿元，引进世界500强企业10 家、上市公司6家。营双高速公路景泰段、吴家川至坝滩公路、沿黄快速通道白桦公路建成；中川机场至白银至刘川工业园公路、黄河石林景区二级公路、靖远至会 宁侯川段公路提升改造工程、平堡黄河大桥开工建设；白银天博万头奶牛、景泰正大现代农牧业产业示范等项目加快实施。城市发展亚行一期项目基本完成，累计投 资12.09亿元，二期项目前期工作进展顺利。总投资40亿元的中和教育港、文化商住综合体等项目签约。

5.园区建设势头强劲。工业集中区总体发展规划获省政府批复，建设用地纳入国家低丘缓坡未利用地开发试点。管理体制逐步理顺，“一区六园”纳入统一管理， 全年完成基础设施建设投资12.75亿元，开发面积达到82.46平方公里。银东工业园纳入城市总体规划；银西工业园基本实现“七通一平”；刘川工业园具 备承载重大项目基础条件；高新区获批为国家知识产权试点园区，在国家高新区排名前移13位，名列72位，科技企业孵化器一期5万平方米生物医药产业园建成 投用，摩尔化工等52家企业入孵，二期装备制造产业园建成5.3万平方米。工业集中区完成固定资产投资201.5亿元，实现技工贸总收入628亿元，同比 增长31.1%；完成工业增加值198.9亿元，同比增长17%。

6.城乡面貌不断改善。城市总体规划编制完成，控制范围由135平方公里扩大到312平方公里，银光公司生活区等4个片区控制性详规初步完成，村镇建设规 划全面启动。实施城建项目83项，完成投资38.6亿元，新建改造国道109线过境段、人民路、长安路、南环路等城区道路17条30.85公里，“六横八 纵”的路网结构基本形成，建成区面积扩大到80平方公里；实施金岭公园、银光公园、西区人民广场改造工程，城市矿山公园博物馆建成，220个公交候车亭建 成投用；开展拆墙透绿、拆违增绿，新增绿地73.94公顷；改造失管小区5个、住宅楼42栋，整治背街小巷23条、2.6万平方米。整合市区集中供热17 万平方米，新建改造管网6.6公里。城市“三维数字社会管理”系统启动建设，我市被列为国家智慧城市试点。会宁、靖远、平川新城区建设步伐加快，景泰文化 广场改造工程全面完成，全市城镇化率提高1.7个百分点，达到43.2%。新建农村公路600公里，基本实现乡乡通油路、村村通公路；新续建集中供水工程 22处，解决了15万人饮水不安全问题。全市完成造林33.18万亩，城区大环境绿化1.36万亩，森林覆盖率达到13.93%。淘汰落后产能138.1 万吨，预计万元GDP能耗下降5.5%，万元工业增加值用水量下降5.35%，市区空气质量优良天数达到334天，创历史最好水平。全面开展美丽乡村建设 行动，龙湾被评为“中国十大最美乡村”。

7.社会事业全面进步。全市民生方面支出85.84亿元，占公共财政预算支出的72.83%。省市确定的各项惠民实事全部完成。教育体育发展水平巩固提 升。新改扩建校舍30.75万平方米、幼儿园87所、职业教育实训基地4个，建成义务教育标准化学校83所，矿冶学院新校区建成投用；4所市属学校下划白 银区管理，白银区义务教育均衡发展率先通过省级验收；解决了10847名进城务工子女、留守儿童和残疾儿童的入学问题；农村义务教育学生营养改善计划惠及 学生11万人；高考二本以上录取人数突破万人。完成体育惠民工程6个、农民体育健身工程77个，成功承办了全省第二届中学生运动会和全省青少年科技创新大 赛。医疗卫生事业稳步发展。新农合筹资标准由人均290元提高到340元，参合率达97%；基本公共卫生服务经费人均由25元提高到30元。市第一人民医 院门诊大楼投入使用，市儿童医院、残疾人康复中心开工建设。免费孕前优生健康检查全面开展，计生基础工作进一步巩固。就业工作持续加强。投入1.14亿 元，开展职业技能培训8.35万人、创业培训3515人；高校毕业生实现就业6995名，城镇新增就业7.67万人；劳务输转31.07万人，劳务收入 39.23亿元。社会保障能力不断增强。城乡居民社会养老保险基础养老金从人均每月55元提高到60元，城乡低保月人均补助水平分别提高10%和 13.5%，农村五保年供养标准提高200元，发放城乡低保金5.35亿元，建成农村互助老人幸福院150所；新开工各类保障性住房21751套，建成 9604套，改造农村危房1.13万户。扶贫攻坚成效明显。落实专项投资1.54亿元，实施整乡整村推进项目34个，减少贫困人口7.33万，贫困面由 52%下降到46%。社会大局和谐稳定。法制宣传教育广泛开展，我市被评为全省“六五”普法中期先进市，矛盾纠纷排查化解和调解联动机制进一步完善；实施 “天网工程”，有效防范和惩治各类违法犯罪活动，主要刑事案件发案率同比下降14%；隐患排查、“打非治违”、安全生产大检查等专项行动扎实开展；全市各 民族团结，宗教和顺。

8.政府建设得到加强。取消行政事业性收费17项，按下限执行6项，取消、调整、合并审批事项49项，推行工业类项目并联审批，全市四级政务服务平台实现 全覆盖。全市政务服务中心、便民服务站点共受理事项128.6万件，办结率99.8%。组建公共资源交易中心，完成进场交易353宗、50.3亿元，节约 资金5500万元。进一步理顺食品药品监督管理体制，完成工商、质监行政管理体制划转移交工作。市级层面会议同期减少39次，发文同比下降14%，信息简 报由150种减少为45种。市本级“三公”经费下降15%。开展效能专项督查73次，督查项目373个，多年受征地拆迁影响的长安路、南环路、国道109 线过境段等项目顺利推进。清理、撤销各类议事协调机构274个，腾退办公用房1.17万平方米。办理行政复议案件16件。办理人大代表建议74件，政协委 员提案180件。扎实推进联扶行动，帮办实事5800多件，协调争取项目2400个，落实资金1.13亿元。

一年来，全市审计、统计、双拥、老龄、粮食、档案、人防、气象、地方志、公积金、残疾人、防震减灾、红十字会等工作都取得新的进步。

各位代表，2013年我市经济社会发展取得了明显成绩，转型发展迈出了新的步伐。这是省委、省政府和市委科学决策、正确领导的结果，是市人大、市政协监督 支持、共识共为的结果，是地企、军地密切配合、通力协作的结果，是全市人民齐心协力、团结奋斗的结果。在此，我代表市人民政府，向全市人民、各民主党派、 人民团体、工商联和社会各界人士，向中央、省驻银各单位，向驻银部队、武警官兵致以崇高的敬意和衷心的感谢！

在肯定成绩的同时，我们也清醒地认识到，经济社会发展和政府工作中还存在一些困难和问题：农业种养加结构不合理，产业化程度不高，工业仍主要集中于资源开 发和初级产品加工领域，第三产业发展缓慢，转方式、调结构、促发展的任务艰巨；产业链招商引进的大项目、好项目还不多，对经济增长的拉动作用不够；交通、 水利、园区等基础设施建设落后，对转型发展的支撑作用不强；改革创新活力不足，企业自主创新能力不强，金融推动转型发展的作用发挥不够；城乡二元结构矛盾 突出，城镇化建设滞后，城乡居民收入水平低，改善民生的任务繁重；工作作风有待转变，一些部门的执行力还需加强，服务发展的能力和水平有待提升。市政府将 逐一分析这些困难和问题，制定切实有效措施，认真加以解决。

2014年目标任务和重点工作

2014年，是贯彻落实党的十八届三中全会精神、全面深化改革的开局之年。政府工作的总体要求是：全面贯彻落实党的十八大和十八届三中全会精神，坚持稳中 求进的总基调，以改革创新为动力，以改善民生为根本，以白银工业集中区为平台，大力发展壮大“3+4”产业集群，大力实施“3341”项目工程和 “1236”扶贫攻坚行动，大力推进循环经济示范区、承接产业转移示范区、华夏文明传承创新区、国家生态安全屏障综合试验区和兰白科技创新试验区建设，加 快基础设施建设步伐，加快新型城镇化进程，加快政府职能转变，切实提高经济增长质量和效益，着力促进经济转型升级、社会和谐稳定。

经济社会发展主要预期目标是：生产总值增长12%，规模以上工业增加值增长15%，固定资产投资增长28%，社会消费品零售总额增长14.5%，公共财政 预算收入增长13%，城镇居民人均可支配收入增长11%，农民人均纯收入增长13%，城镇登记失业率控制在4%以内，人口自然增长率控制在7.2‰以内， 居民消费价格总水平涨幅控制在3.5%以内，单位生产总值能耗和主要污染物排放总量控制在省上下达的目标以内。

今年，重点抓好以下七个方面的工作：

一、全面推进改革创新，不断增强发展活力

切实转变政府职能。做好审批职能“接、放、管”工作，建立权力清单和负面清单制度，编制“行政审批事项目录”，推进发改、住建、国土、环保等部门审批事项 同步下放、并联审批。完成政府机构改革，组建不动产登记管理机构，启动建设登记信息管理平台；优化工商、质监管理体制，整合卫生、人口等领域职能，理顺市 区城建规划、市政管理、市场监管和食品药品执法职责。进一步规范部门责任主体，清理各类行政事业性收费。全面推行事业单位绩效管理，完成事业单位分类工 作。整合公共资源交易项目，推行电子招标采购，确保各类交易业务全部上线。调整完善考核指标体系，实行县区差异化考核，加大约束性指标和社会发展指标权 重，强化考核结果应用。

大力发展非公经济。编制非公经济发展纲要，落实好优惠政策，废除各种不合理规定，鼓励非公经济参与国有企业改革，投资铁路、公路、城镇、园区等基础设施建 设，进入公共服务领域；加快中小微企业服务体系建设，推动非公企业建立现代企业制度，推动产权结构由单一化向多元化、社会化转变，组织形式由个人业主制、 合伙制向混合所有制转变，管理模式由家族式向法人治理结构过渡；改进资信评估制度，健全信用担保体系，推动上市融资；推进工商登记制度改革，推行先照后证 登记制、注册资本认缴制、年度报告制，实现非公企业户数增长16.5%，非公经济增加值增速达到22%。

加快国有企业改革。推动市属城投公司、水务集团、交通建设公司、黄河旅游公司等政府类投融资企业和供热、供水、公交等公益性企业政企分开、规范管理，建立 完善薪酬和资产经营责任制度；推动白银公司、靖煤公司、稀土公司等国有大中型企业股份制改造，完善现代企业制度。

健全完善市场体系。建立统一的生产要素市场准入制度，实现市场主体依法平等进入。建立城乡统一的建设用地市场，农村集体经营性建设用地与国有土地同等入 市、同权同价。深化资源性产品价格改革，制定资源市场改革具体意见，推进大用户直购电直接交易试点；推进水权制度改革、农业水价综合改革，在景电、靖会、 兴电等大中型灌区推行终端收费和超定额累进加价制度；建立和完善城镇居民阶梯水价、气价制度。

深化财税金融改革。坚持先有预算、后有支出原则，科学合理编制预算，建立跨年度预算平衡机制，集中财力优先保障民生支出，扶持重点产业加快发展；稳步推进 预决算公开，提高财政运行的透明度；将地方政府性债务纳入预算管理，建立联席会议、年度审计、责任追究等制度。进一步推进农村金融创新改革工作，做好“三 权”确权、发证及贷款投放工作，继续开展信用乡镇、信用村创建工作，优化金融生态环境；设立政府引导基金，吸引各类金融机构、创业投资机构入驻，推进中银 富登发起设立平川村镇银行；构建普惠金融体系，加强对贫困人口、低收入群体和小微企业的金融服务；建立信贷投放与财政性存款挂钩机制，鼓励扩大信贷规模， 新增信贷投放60亿元以上。

创新园区管理建设机制。推行“管委会、经济实体、社会中介组织”三位一体的园区开发模式，行政管理主体与开发建设主体相分离，实现经济行为实体化、园区建 设市场化、公共服务社会化。设立工商、地税、公安、安监等职能机构，推行扁平化管理。建立园区企业担保资金和企业联保机制，争取高新发展公司通过评级认 证，拓宽融资渠道。引进金融、法律、评估、审计等各类专业化中介服务机构，规范发展行业协会、商会等自律性组织。设立产业投资基金，探索创新知识产权质押 融资、信用贷款、中小企业集合债券等金融产品，运用市场化机制，加快园区配套设施建设。全年工业增加值增长17%以上，固定资产投资增长30%。

持续推进科技创新。加强与高等院校和科研机构产学研合作，成立高新区科技成果转化机构，规划建设科技成果转化园中园和中试产业基地，鼓励科研人员以技术入 股、股权激励等形式参与产品研发和成果转化。加强同省科技发展投资公司合作，利用省新材料和环保产业等国家创业投资基金平台，完善多元化科技投融资体系。 启动建设兰白科技创新综合配套改革试验区，推进国家知识产权试点城市建设，加速科技成果转化，培育技术交易市场，新增高新技术企业3家、科技型企业15 家，专利申请受理量增幅达到30%，突破500件，万人发明专利拥有量达到1件。

二、加快转变发展方式，促进产业转型升级

加快发展现代农业。全面落实“365”现代农业发展行动计划，深入实施粮安工程，突出发展玉米和马铃薯两大高产作物，确保粮食总产稳定在72万吨以上；推 广新型农作制度，形成亩产“千斤粮、万元钱”农业发展模式，带动农民收入持续增加。新改扩建日光温室1万亩、塑料大棚0.6万亩，种植特色林果13万亩， 发展粮草兼顾的草地农业，新增标准化规模养殖场70个、规模奶牛场5个，做好动物疫病防治工作。新增龙头企业10户、专业合作社210家，培育壮大瓜菜、 枸杞、小杂粮、羊羔肉等优势农产品加工企业，加工转化率达到44%。加快实施引洮一期会宁北部供水、兴电灌区齐家大岘除险加固、百万亩节水等工程，确保双 永供水工程主干渠通水运行，新增有效灌溉面积2.5万亩，兴修梯田12万亩，完成水土保持综合治理120平方公里，解决10万农村人口饮水不安全问题。抓 好农村土地确权登记试点工作，建立农村产权交易所，促进农民土地承包经营权入股新型经营主体，保障农民对承包地占有、使用、收益、流转及承包经营权抵押、 担保权利，扶持土地流转大户50户，土地流转面积达到50万亩。抓好农产品质量安全，创建农业标准化示范基地、标准化养殖示范场10个，扩大“三品一标” 农产品覆盖面。

推动工业提质增效。加快实施一批延链补环项目，进一步壮大骨干企业、主导产业，壮大“3+4”产业集群。做强能源及高载能产业，抓好热电联产、鸿泰铝合金 产业链、国电靖远发电“上大压小”、华鹭铝业异地搬迁扩能等项目推进，开工建设中凯景泰寺滩风电、大唐前台子风电、甘肃德祐靖远光伏发电等新能源开发项 目，新增装机容量40万千瓦。做深有色产业，加快白银公司铜冶炼技术提升改造、锌冶炼资源综合利用、稀土新材料园、郝氏炭纤维炭复合新材料、银泰铝业板材 深加工等项目建设，进一步提高产品深加工率和新材料产业比重。做精化工产业，推进25万吨硝基复合肥二期、东方钛业钛白粉等项目建设，抓好银光集团TDI 二期、刘化煤制气等项目的论证立项，促进煤电联动，大力发展煤化工产业。做活百亿元产业，陶瓷建材产业抓好凯斯瓷业陶瓷微粉抛光砖、泰山石膏板、富海阳光 建材项目建设，加快住房新型材料示范推广，建成平川陶瓷产品省级质检中心；先进装备制造产业抓好中凯风电装备制造、容和矿用电器技术改造、甘肃倚银石化装 备制造等项目，推动新能源配套装备产业发展；生物医药产业重点实施赛诺生物酶、正生生物籽瓜废弃物综合利用、酵母饲料生产等项目，引进中药产业化企业，加 快生物经济园建设。强化工业企业运行监测分析，加强重点行业、重点企业协调调度，保障煤、电、油、运等生产要素供应，做好中小企业入规培育，提高工业经济 运行质量和效益。

着力发展现代服务业。制定大物流产业发展扶持政策，加快推进大西北仓储式商贸物流园、现代工业物流、环银百大楼商贸城等项目，打造银东工业、银西商贸两个 核心物流园区，构建公铁航联运物流体系。抓好平川忠恒金地年鲜农贸市场、靖远西北果蔬冷链物流中心、县乡农贸市场标准化提升改造、万村千乡市场工程，建设 雒家滩废旧物资交易市场、郝家川水产品批发市场、长安路综合农贸市场，组建中小商贸流通企业服务中心，大力发展电子商务，扩大信息消费，建设国家中小商贸 流通企业服务体系试点城市。抓好“新网工程”，推进基层供销网络建设。大力发展健康服务业和养老服务业，吸引民间投资，推动四龙养老服务中心建设。

三、着力保障和改善民生，不断增进人民福祉

健全社会保障体系。强化社会保险扩面征缴工作，拓展社会保险参保覆盖面。加强农村低保与扶贫开发“两项制度”的有效衔接，农村五保供养标准提高510元。 继续推进农村互助老人幸福院建设，开展城市社区居家养老服务和农村留守老人日间照料帮扶工作。全面落实地方储备粮承储计划，严格执行灾情监测和值班制度， 不断增强抵御灾害风险能力。新建各类保障房6546套，改造农村危旧房1万户，为9491户发放廉租房补贴2887万元。

大力发展社会事业。持续推进全民创业行动，加强对高校毕业生、就业困难人员的就业指导服务，新增城镇就业6.2万人。全力推进义务教育均衡发展、高中教育 特色发展，新改扩建校舍10万平方米，消除危房10万平方米，建设义务教育阶段标准化学校100所。大力发展学前和职业教育，加强幼儿师资力量，完成矿冶 学院招生1500人。不断深化医药卫生体制改革，开工建设市第一人民医院住院部大楼，加快中医药制剂中心建设，建成乡镇卫生院30个。新农合筹资标准由人 均340元提高到390元。全面落实计生各项奖励优惠政策，贯彻执行单独两孩生育政策，促进人口长期均衡发展。大力开展群众体育运动，全力办好甘肃省第十 三届运动会和第九届残疾人运动会。继续抓好质量振兴工作。全面做好第三次经济普查。

改进社会治理方式。建立党组织、居委会、工作站、服务站“四位一体”的社区工作格局，将社区承担的行政事项逐步转移到社区工作站。全面推行政府购买公共服 务，推进社会组织与行政机关脱钩，做好行业协会商会类、科技类、公益慈善类、社区服务类等社会组织直接登记工作。建立网上受理信访事项和群众满意度评价体 系，健全司法救助制度、社会稳定风险评估机制和矛盾纠纷预警、联动、对接、调处化解综合机制，完善立体化社会治安防控体系，推进“平安白银”建设。建立严 格的食品药品安全监管和责任追究制度，提高食品药品安全保障水平。全面落实“党政同责、一岗双责、齐抓共管”的责任体系，确保安全生产形势持续稳定好转。

强力推进扶贫攻坚。深入实施“1236”扶贫攻坚行动，促进扶贫工作与“双联”行动的深度融合。推动移民搬迁“下山入川”、“出村入城”，减少贫困人口 7.19万；着力完善专项扶贫、行业扶贫和社会扶贫“三位一体”大扶贫格局，统筹抓好3个集中连片特困县和2个插花贫困区扶贫工作。整合培训资源，加大订 单培训和劳务输转力度，拓宽增收渠道。

兴办9件惠民实事：（1）实施扶贫攻坚整村推进项目20个；（2）建成基层文化大院60个；（3）建成标准化村卫生室25个；（4）提升改造农贸市场10 个；（5）建成“一村一场”农民体育健身工程50个；（6）实现乡镇公办中心幼儿园全覆盖、改建农村小学附设幼儿园100所；（7）改造失管小区5个； （8）补贴援助办案经费，为困难群众提供法律援助服务；（9）建设全市乡村道路交通安全服务点。

四、全力抓好项目工程，增强区域竞争优势

对接国家政策谋划项目。实行项目谋划责任制，充实完善项目库。围绕全省建设全国重要的新能源及新能源装备制造基地，谋划实施一批新型储能、现代高载能、二 次电池及电池材料等项目；围绕全省建设新能源外输工程，谋划布局电源点，探索分布式能源发展和新能源局域电网建设；围绕“丝绸之路经济带”建设，谋划实施 一批矿产资源开发、农产品加工、文化旅游等合作项目；抢抓国家支持现代服务业发展的机遇，谋划发展生产性服务业和生活性服务业；结合全省煤化工产业发展规 划，谋划一批煤炭分质转化利用项目；用足用活国家重点支持战略性新兴产业政策，谋划一批生物医药、信息、节能环保等项目；主动对接国家生态安全屏障综合试 验区建设，谋划实施一批水源涵养、流域保护、风沙治理等生态项目；研究新一轮国家棚户区改造政策，争取专项资金，完成50万平方米棚户区改造任务。

围绕产业链条引进项目。充分利用兰洽会、民企陇上行等平台，紧盯500强企业，对接“3+4”产业链，开展点对点精准招商。能源产业大力引进光热发电技 术，建设光热发电及储能新材料示范园，推进煤电铝一体化；化工产业以TDI产业链为核心，重点引进煤制天然气、煤制甲醇、煤制烯烃等项目，推进煤化工和 TDI上下游产业融合，建设西部重要的煤化工和精细化工基地；有色产业重点引进铜铝铅锌合金材料、功能材料、复合材料、高纯材料等项目，建设有色（稀土） 新材料基地；先进装备制造产业重点引进风电装备、光伏发电设备、矿山工程机械、农机机械等项目；陶瓷建材产业重点引进特种陶瓷、凹凸棒开发、玻璃制造等项 目；资源循环利用产业着力引进煤矸石发电、稀贵金属回收利用等项目；生物医药产业重点引进生物制药、生物制品、生物能源、生物育种等项目。

突出基础设施推进项目。完善领导干部包抓重大项目责任制、项目建设联席会议制度，推行“管家式”、“一站式”服务，推动项目顺利实施。对前期工作比较完备 的干塘至武威南增建二线、景泰至会宁二级公路、沿黄快速通道、黄河上游白银段防洪治理堤防工程等重大项目，加大工作力度，确保动工实施；对事关我市长远发 展的甘肃中部生态移民扶贫开发供水工程、包兰铁路扩能改造、庆阳环县经白银至兰州新区铁路、白银至平凉铁路等项目，成立专门机构，推进前期工作；加快推进 白银至中川机场、景泰至黄河石林等公路建设；抓住国家路网干线升级改造机遇，启动3条省道升国道改扩建工作，争取10条县乡道列入省道网规划。加快白银工 业集中区建设，高新区以打造国家级科技企业孵化器为目标，提高一期运营管理水平，加快二期建设进度，开工建设三期；银东、银西工业园重点推进水、电、通讯 等配套工程，实现园区与主城区有效对接；刘川工业园重点抓好亚行二期项目实施，加快给排水管网、道路硬化、绿化等基础设施建设；正路工业园实现主干线路、 给排水管网全面贯通；平川工业园加快南区开发；会宁工业园重点推进西城产业开发区基础设施建设。

五、推进文化旅游融合发展，建设华夏文明传承创新区

加快文化事业建设步伐。推进县区“文化集市”建设，扶持培育“乡村舞台”，形成市、县（区）、乡（镇）、村（居）四级公共文化服务网络，抓好“历史再现” 工程和农村数字电影放映工程。推进市文化艺术中心、国际青少年儿童美术博览馆和地震博物馆等文博设施建设，抓紧实施永泰城址保护维修工程。扩大图书流通站 建设规模，开通运行手机移动图书馆、电视图书馆业务。

加大文化旅游产业开发力度。加强与省公航旅集团、康辉集团的合作，加快黄河石林、黄河假日城等景区开发建设进度，谋划建设黄河大峡谷风景旅游区，协同兰 州、临夏两市推进黄河风情线大景区建设，打造丝绸之路沿线文化旅游带。抓好全国工商联水产业商会靖远文化产业开发、新天地文化商业街三期、会宁汉唐二十四 节气文化休闲商业街、铜艺陶艺开发和县区数字影院等文化项目。新建改造标准化“农家乐”25家。组建市文化传媒公司和演艺公司，建成2个以上游客服务中 心，加大特色文化旅游产品开发扶持力度，文化产业增加值增长28%。

加紧建设华夏文明传承创新区。抓好红色文化产业园、黄河石林旅游文化产业园、影视文化产业基地、青少年艺术培训基地、黄河民俗文化产业带、矿业文化博览 园、陶瓷文化产业园、铜文化产业园等产业基地建设，吸引更多企业入驻。深入挖掘黄河古渡、彩陶、大爆破、电力提灌工程等历史现实题材，拍摄电影《羊皮筏 子》，创编歌舞剧《梦幻彩陶》，推出一批精品力作。

六、坚持生态文明理念，推进“两型”社会建设

更加注重循环发展。抓好国家循环经济示范城市建设，加大西北再生资源产业基地推进力度，重点实施废旧汽车拆解、生活垃圾焚烧发电、废旧电池回收利用等项 目，争取我市列入国家“城市矿产”示范基地。加快高新区、银西工业园、平川工业园循环化改造，确保完成60%以上改造任务。继续实施合同能源管理和既有建 筑节能改造，培育循环型社会消费方式。建立健全废旧农膜回收利用体系，回收率达到77%；推广农作物秸秆还田、生物堆肥、加工饲料等综合利用技术，农作物 秸秆综合利用率达到80%。

更加注重环境治理。加快东大沟河道整治、重金属点源深度污染治理。开展矿山复绿行动，整治矿山地质环境。继续实施工业脱硫、脱硝、锅炉改造和农业源减排工 程，推进大气环境持续改善。严格落实国家强制性能耗标准，淘汰落后产能44.34万吨，争取列入国家节能减排财政政策综合示范城市；加强有色、化工、水 泥、硅铁、电石等行业监管，确保达标排放。

更加注重生态保护。加快国家生态安全屏障综合试验区建设，重点实施农田林网、林缘区造林、绿色通道、荒山造林、大环境绿化等工程，启动中信集团碳汇林建设 项目，完成造林32万亩。争取将三县两区纳入新一轮退耕还林范围和退牧还草项目区。探索建立生态保护与补偿新机制，落实草原生态保护补助奖励、退耕还林补 贴、土地复垦、垃圾处理收费等政策。

更加注重乡村美化。继续开展美丽乡村建设行动，巩固扩大“四清”和“四化”成果，完成景电、兴电、靖会等高扬程提灌区15个乡镇168个村的环境治理；开 展农村山坡地、废弃闲置宅基地整治；在条件较好的村镇，建立公益性公墓或集中安葬区；成立乡镇环卫所和村保洁站，建设无害化垃圾处理设施，实行环境卫生考 评，确保农村环境面貌明显改观。

七、加快新型城镇化建设，构建城乡一体发展格局

优化城镇布局。对接国家新型城镇化规划、省域城镇体系规划，完成全市新型城镇体系规划，加快推进兰白一体化进程，积极融入兰州-西宁国家城市群建设，构建 “一城三带三中心”城镇体系布局。中心城区以“揽河入城、纳山为屏”为目标，依托白银高新区、银西工业园、银东工业园，进一步完善功能，加速产业和人口集 聚，形成产城互动、城乡一体复合型城镇密集区；以四龙、水川滨河新镇建设为重点，打造集文化、旅游、休闲、人居于一体的城市拓展区，加快城区绿化和水系建 设，让市民望得见青山、看得见绿水。建设联接水川、四龙、北湾、糜滩、三滩、东湾、水泉、龙湾、五佛等重点村镇的沿黄城镇带，联接白银、刘川、王家山、北 滩、东升、五合的刘白高速城镇带，联接景泰、平川、靖远、会宁的国道247线城镇带，着力打造郭城、北滩、红水三个县域经济次中心。

完善基础设施。白银城区重点新建上海路北段、银西中路等道路8条，续建北环路、国道109线过境段，改造北京路东段、广场东路等道路4条；集中抓好银东、 银西工业园供水管网和旧城区供水管网改造；开工建设银西工业园热源厂和热电联产供热管网，推进平川、靖远热电联产集中供热工程，逐步实现城市供热集中化； 继续实施金岭公园改扩建、银凤湖公园续建、银光公园二期、西山公园改造、矿山公园景观建设等项目，建设东部城乡枢纽站、东入城公园、北城区人工湖；重点抓 好中心街、八号楼等片区改造，实施刘家梁、大井子、黄茂井、周家地等城中村改造，建设城市新型社区。建设农村公路500公里。加快实施数云白银云计算产业 园项目，做好“宽带提速”工程，推进“智慧城市”建设，提高城乡信息化水平。

加强城镇管理。结合“五城联创”，加大市容市貌、城区污染、交通秩序和社会治安等综合治理力度，提高居民综合素质，提升城市整体形象。城市管理重心向街道 和社区下移，加强建制镇、社区管理，规范小区物业管理，推行生活垃圾移动收集和道路保洁责任制。加强城市规划管理，推行城市红线、绿线管理。强化工程建设 管理，严把设计、施工、监理、竣工验收关口，确保工程质量。

统筹城乡发展。制定完善农业转移人口及其子女就业就学、购房落户、社会保障等政策，推进农业转移人口市民化，确保城镇化率提高1.7个百分点；以中心村为 重点，加快新型农村社区建设，强化公共服务保障，吸引农民集中居住。全市确定6个建制镇开展新型城镇化试点，争取1个县、两个镇纳入全省新型城镇化试点。

各位代表，全面完成今年的各项目标任务，政府肩负着重大而艰巨的责任，承载着全市人民的厚望。我们将切实转变政府职能，忠实履行职责，攻坚克难，锐意进取，以为民、务实、清廉的作风，不断提高政府的执行力和公信力。

改进作风，科学理政。认真开展党的群众路线教育实践活动，严格执行中央八项规定和省、市委相关规定，聚焦“四风”抓整改，重拳整治“庸懒散”，着力解决 “中梗阻”，努力做到向群众承诺的事全力办，对群众期盼的事加快办，受群众关注的事公开办，尽心竭力做好打基础、利长远的工作，千方百计办好顺民心、惠民 生的实事。进一步健全行政决策机制，完善公众参与、专家论证、风险评估、合法审查和集体讨论相结合的科学决策制度，认真办理人大代表建议和政协委员提案， 确保政府各项决策的科学性、民主性和实效性。

强化执行，高效施政。把执行力作为政府工作的生命线，作为检验干部作风的试金石，全面贯彻落实市委决策部署，强化勇于担当的责任意识，树立一抓到底的执行 精神，保持勤勉奋发的工作状态，咬定目标不放松，一张蓝图绘到底，一年接着一年干，全力推动工作落实。加强行政效能监察，健全督查联动机制，严格执行岗位 责任制、服务承诺制、限时办结制、首问责任制和失职追究制，推行“两集中三到位”政务服务，整合审批部门，再造审批流程，压缩审批环节，减少审批时间，以 高效率赢得高效益，以快节奏换来快发展。

规范行为，依法行政。深入开展“六五”普法工作，进一步培养法治精神、法治素养和法治观念，加强重点领域基层执法力量，严格按照法定权限和程序行使权力、 履行职责，依法公开权力运行流程，规范自由裁量权，推行行政执法责任制和评议考核制，建设法治政府。强化网上政务服务和电子监察平台建设，建立百姓问政制 度，加大群众网上诉求办理力度，推进决策公开、管理公开、服务公开、结果公开，努力做到让人民监督权力、让权力在阳光下运行。

勤政为民，廉洁从政。推进节约型机关建设，改进文风会风，精简会议文件，规范办公用房，严控“三公”经费和一般性支出，将有限的财力集中到惠民生、促发展 上，政府过紧日子、群众过好日子，确保公款姓公、公权为民。健全惩治和预防腐败体系，认真落实党风廉政建设责任制，强化行政监察和审计监督，加强对财政资 金使用、重大项目投资、工程招标、政府采购等重点领域监管，切实解决以权谋私、与民争利和损害群众利益的问题，做到干部清正、政府清廉、政治清明。

各位代表，站在新的起点上，转型跨越的责任和使命激励着我们，破冰前行的困难和挑战考验着我们，人民对幸福生活的追求和期盼鞭策着我们。让我们在市委的坚 强领导下，以前所未有的激情和信心，团结奋斗，真抓实干，做厚德勤奋的白银人，建宜居宜业的白银城，走改革创新的白银路，圆和谐幸福的白银梦，共同开创白 银更加美好的明天。

术 语 解 释

三权：即农村土地承包经营权、宅基地使用权和林权。

“三维数字社会管理”系统：即以建设市级统一的社会管理服务体系为目标，以统一的三维地理信息系统、人口法人数据库、视频实景监控为基础，建立受理平台、 呼叫中心、应急处置、在线办事等市、区、部门、街道、社区、楼院六级联动、集中统一的社会服务中心，协同办公的工作模式。

智慧城市：即把新一代信息技术充分运用在城市的各行各业之中的基于知识社会下一代创新的城市信息化高级形态。

天网工程：即公安部联合工信部等相关部委共同发起建设的利用图像采集、传输、控制、显示等设备和控制软件组成，对固定区域进行实时监控和信息记录的视频监控系统。

打非治违：即打击非法、违法生产，治理违规、违章作业简称。

“3+4”产业集群：即“十二五”期间，我市重点规划打造的有色金属（稀土）深加工及新材料、化工、能源和先进高载能3个千亿元产业链，先进装备制造、陶瓷建材、农畜产品深加工、生物医药4个百亿元产业。

“3341”项目工程：即打造以兰州新区开发建设和循环经济示范区建设、华夏文明传承创新区、国家生态安全屏障综合试验区为重点的经济、文化、生态三大战 略平台，实施交通提升、信息畅通、城镇化三大基础建设，瞄准战略性新兴产业、特色优势产业、富民多元产业、区域首位产业四大产业，力争到2016年全省固 定资产投资规模达到1.5万亿元。

“1236”扶贫攻坚行动：即围绕“一个核心”和“两不愁、三保障”，全力实现“六大突破”。“一个核心”就是持续增加贫困地区农民收入，“两不愁”就是 不愁吃、不愁穿，“三保障”就是落实义务教育、基本医疗和住房三个保障，“六大突破”就是实现基础设施建设、富民产业培育、易地扶贫搬迁、金融资金支撑、 公共服务保障、能力素质提升六个方面的突破。

“365”现代农业发展行动计划：即着力打造旱作农业、高效节水农业和草原畜牧业可持续发展三个国家级示范区；壮大和提升六大特色优势产业，壮大以草食畜 牧业为主的循环高效农业、以设施蔬菜为主的高效节水农业、以优质林果为主的区域性特色高效农业，提升马铃薯、中药材、现代种业和酿酒原料等特色产业；强化 农业设施装备和科技创新、农村基础设施建设、新型农业生产经营主体培育、农民职业技能培训、政策支持和保护五大支撑。

“千斤粮、万元钱”农业发展模式：即通过种养结合、粮经复合、水旱轮作及循环利用，科学配置土地、物种、时空，充分挖掘农田生产潜力，增加复种指数，提高 农业综合生产能力，促进粮经作物协调发展，构建优质高产、节本增收、安全生态的高效农作模式，确保实现每亩年产“千斤粮、万元钱”。

三品一标：即无公害农产品、绿色食品、有机农产品和农产品地理标志的统称。

新网工程：即全国供销合作总社“新农村现代流通服务网络工程”的简称。

文化集市：借用了“赶集”的概念，即在固定的地点、固定的时间演出不同的文艺节目。

“历史再现”工程：2014年全省文化局长会议上正式提出，即通过推动重大文物保护项目的实施，展示各类不可移动文物的价值和内涵，拓展可移动文物的展示 手段，创新博物馆建设的机制体制，进一步加大文化遗产保护力度，促进文化遗产合理利用，进一步提升和显现甘肃文化优势。

四清：即在我市美丽乡村建设行动中开展的清垃圾、清杂物、清残垣、清庭院活动。

四化：即在我市美丽乡村建设行动中开展的净化、绿化、亮化、美化活动。

五城联创：即在全市范围开展创建全国双拥模范城市、国家卫生城市、国家环保模范城市、省级园林城市和全国文明城市的活动。

两集中三到位：即为深化行政审批制度改革，建立规范高效的审批运行机制，提高行政服务效能，推进一个行政机关的审批事项向一个处室集中、行政审批处室向行政审批服务中心集中，保障进驻行政审批服务中心的审批事项到位、审批权限到位、审批人员到位。
